# Why health diplomacy should be understood as a deterrence instrument: evidence from Sweden’s health system

**DOI:** 10.1186/s12992-026-01210-2

**Published:** 2026-04-14

**Authors:** Matteo Maria Cati

**Affiliations:** 1 Division of Health Research, Lancaster University, Lancaster, UK; 2 Department of Economics, University of Bologna, Bologna, Italy

**Keywords:** Health diplomacy, Globalisation and health, Health security, Antibiotic shortages, Pharmaceutical supply chains, Antimicrobial resistance, Nordic cooperation, European Union, Health system resilience, Health deterrence

## Abstract

Sweden’s health system is increasingly exposed to strategic vulnerabilities arising from globalised pharmaceutical supply chains, medicine shortages, antimicrobial resistance, and hybrid geopolitical threats. In a period of heightened international tension, safeguarding health security has become an urgent policy concern. While the securitisation of health diplomacy raises ethical and political challenges, this article argues that health diplomacy should be understood not only as a cooperative instrument but also as a form of deterrence that operates through governance integration, transparency, and strategic interdependence, strengthening national resilience. In this context, deterrence refers to reducing the incentives and opportunities for adversarial actors to exploit health-system vulnerabilities, such as pharmaceutical shortages or supply-chain disruptions, to generate societal disruption, undermine public trust, or exert political pressure. Focusing on Sweden, the article shows how deeper Nordic and European Union coordination enhances anticipatory capacity, stabilises access to critical medicines, particularly antibiotics, and reinforces crisis communication and public trust. Positioning health diplomacy within broader security architectures can support continuity of care during systemic stress while remaining anchored in civilian governance and global health norms.

## Introduction

Across Europe, health systems are increasingly recognised not only as public goods but also as strategic infrastructures exposed to geopolitical tensions, hybrid interference, and globalised market structures. For small or open health systems such as Sweden’s, structural fragilities, ranging from medicine shortages to limited surge capacity, have implications that extend well beyond clinical performance. Recent disruptions affecting pharmaceutical supply chains [3, 4], antimicrobial availability [5–7], antimicrobial resistance (AMR) vulnerabilities [8, 9], hospital and intensive care unit (ICU) capacity pressures [10, 11], elective-care waiting lists [12, 13], and cross-border emergency preparedness [14, 15] illustrate how health-system instability can be amplified by both unintentional market dynamics and deliberate hybrid threats [16, 17]. Taken together, these developments underscore the need to reconsider health diplomacy not only as an instrument of cooperation, but also as a component of national security and deterrence within Sweden’s evolving strategic landscape.

This article advances a deliberately affirmative position, arguing that health diplomacy should be explicitly understood as a deterrence mechanism embedded within national and regional security architectures, while critically engaging with counterarguments concerning securitisation, equity, and the politicisation of public health.

Specifically, this debate article contends that Sweden’s health security would be strengthened through more coordinated domestic governance and deeper integration within Nordic and European Union preparedness frameworks. Drawing on emerging evidence, it argues that governance integration enhances health-system resilience against hybrid threats, systemic shocks, and transboundary crises [27].

By conceptualising health diplomacy as a form of deterrence grounded in interdependence rather than coercion, this article contributes to ongoing debates in globalisation and health governance on how states can manage vulnerability in highly interconnected systems.

In this context, deterrence refers specifically to reducing the incentives and opportunities for adversarial actors to exploit health-system vulnerabilities, such as pharmaceutical shortages, supply-chain disruptions, or hospital capacity constraints, to generate societal disruption, undermine public trust, or exert political pressure. This deterrent effect does not operate through coercion, but through institutional interdependence: by embedding national health systems within transparent, coordinated, and mutually reinforcing governance frameworks, the potential impact of disruption is reduced, thereby lowering its strategic attractiveness.

This debate article adopts Sweden as a strategically relevant case of a small, open, and highly interconnected health system. It draws on policy documents, regulatory sources, Nordic and EU preparedness frameworks, and recent scholarship on health-system resilience, shortages, and hybrid threats. The analysis is interpretive and policy-analytical rather than causal in a strict empirical sense, and aims to assess how governance integration may strengthen resilience and deterrence under conditions of systemic vulnerability, drawing on established frameworks for assessing health-system resilience [19 –21].

## Sweden’s structural vulnerabilities in a changing security context

Sweden maintains a high-performing, equitable health system [18], yet several structural features limit its resilience under persistent stress. The country relies heavily on imports for pharmaceuticals and medical countermeasures, and domestic production capacity remains modest. Shortages of antibiotics, paediatric medicines, cardiovascular drugs, and oncology treatments have become increasingly common [3, 4]. Comparable patterns are documented across Norway, Denmark, and Finland [5–7], indicating regional exposure to global manufacturing concentration and supply-chain fragility. Staffing constraints persist, particularly in rural regions, and Sweden’s per-capita ICU bed capacity remains below many European comparators [10, 11]. Elective-care backlogs have remained well above pre-pandemic levels, reducing system flexibility during emergencies [12, 13].

Geopolitically, hybrid threat assessments conducted by the Swedish Civil Contingencies Agency (MSB) and Nordic security institutions identify health infrastructure as a potential target for destabilization [24–26]. The 2024 Hybrid CoE Working Paper highlights that adversarial actors may exploit health-system bottlenecks, such as medicine shortages or hospital capacity constraints, to generate public concern or erode institutional trust [27]. Sweden’s geographic role at the intersection of Nordic, Baltic, Arctic, and EU security environments adds complexity to these threat vectors [22, 23].

These dynamics point toward the need for a coherent, diplomatically grounded resilience strategy capable of addressing both clinical and security-related vulnerabilities. In particular, the persistence of such vulnerabilities increases the potential effectiveness of hostile or opportunistic disruption, thereby elevating their strategic relevance. Reducing these vulnerabilities through coordinated governance and international integration is therefore not only a matter of resilience, but also a mechanism of deterrence, as it limits the potential impact and attractiveness of interference.

## Medicine shortages as a strategic vulnerability

Antibiotic shortages illustrate how globalization-related processes shape national health system vulnerability. Concentrated pharmaceutical manufacturing, globally fragmented supply chains, dependence on transnational logistics, and regulatory asymmetries mean that disruptions originating far beyond national borders rapidly translate into domestic health system stress. In this sense, antibiotic availability represents a globalization-mediated risk, where local resilience is inseparable from international governance and diplomatic coordination.

Pharmaceutical shortages represent one of the most visible and persistent structural vulnerabilities within Sweden’s health-security landscape. The Swedish Medical Products Agency (MPA) maintains one of Europe’s most comprehensive shortage-monitoring platforms: a publicly accessible catalogue updated daily and covering all reported shortages since 2018 [1]. This system includes real-time information on forecasted start and end dates, confirmed resolution dates, causes (where companies consent to disclosure), authorised substitutes, market-withdrawal notifications, and exemption decisions. Because the catalogue is populated through mandatory reporting by marketing authorisation holders, it provides a detailed, package-level picture of systemic fragility.

Shortage notifications frequently cluster around winter months, especially for antibiotics, paediatric medicines, and respiratory treatments [2, 3]. Forecast revisions occur regularly as companies adjust their expectations, signalling instability in manufacturing or distribution chains. The catalogue’s “Försäljning upphör” designation, indicating permanent market cessation, reflects a further erosion of national medicine availability. Importantly, the MPA notes that not all supply disruptions are reported; medicines may be unavailable despite the absence of a formal shortage notification. This underscores the opacity inherent in global pharmaceutical supply chains and the difficulty clinicians face when trying to anticipate disruptions.

The security implications of these shortages are significant. Nordic regulatory agencies have documented that medicine shortages increasingly arise from global production concentration, just-in-time logistics, limited regional redundancy, and economic disincentives for low-volume markets [4–6]. For Sweden, whose population and whose aggregate market size remains relatively modest in global terms, the risks of supply discontinuity are magnified. Shortage-driven substitution practices also interact with antimicrobial resistance: when narrow-spectrum antibiotics are unavailable, clinicians may be compelled to prescribe broader-spectrum alternatives, accelerating AMR trends [7, 8], thereby amplifying both clinical risk and long-term systemic vulnerability.

Addressing pharmaceutical insecurity requires coordinated action beyond national borders. Nordic collaboration mechanisms, shared monitoring, joint procurement dialogues, coordinated shortage response, and synchronized buffer-stock planning, offer complementary avenues for reinforcing resilience [9–12]. Similarly, Sweden’s participation in EU initiatives such as HERA and the Joint Procurement Agreement expands access to diversified suppliers, pooled stockpiles, and early-warning systems. Pharmaceutical security thus becomes not merely a domestic operational challenge but a domain in which health diplomacy actively contributes to deterrence by strengthening interdependence, transparency, and mutual reinforcement across the region.

For example, a disruption affecting antibiotic supply chains, whether due to manufacturing concentration, logistical failure, or targeted interference in distribution systems, could rapidly generate clinical shortages during periods of peak demand.

In a fragmented system, such disruption could translate into delayed treatments, increased use of broad-spectrum antibiotics, and heightened public concern. By contrast, within an integrated Nordic–EU framework, early-warning systems, joint procurement mechanisms, and shared contingency stocks would both mitigate the immediate impact and reduce the strategic effectiveness of such disruption.

In this sense, interdependence not only enhances response capacity but also acts as a deterrent by limiting the potential consequences and attractiveness of interference.

The strategic importance of antibiotic shortages has recently been recognised at EU level. In December 2025, EU Member States and the European Parliament reached a preliminary agreement on the revision of EU pharmaceutical legislation, explicitly preserving strong regulatory incentives for the development of priority antibiotics, including a voucher-based mechanism. Sweden played an active role in advocating for these provisions, emphasising the link between innovation incentives, equitable access, and security of supply. This agreement represents the first major overhaul of EU pharmaceutical legislation since 2004 and signals a shift towards treating antibiotic availability as a matter of strategic resilience rather than solely a market or clinical issue. By embedding incentives for antibiotic development within EU-wide regulatory frameworks, the agreement illustrates how health diplomacy can operate as a deterrence mechanism, reducing systemic vulnerability through collective governance and market stabilisation.

## Health diplomacy as a strategic deterrence instrument

Health diplomacy has traditionally emphasised cooperation, solidarity, and global public health objectives. Yet in the contemporary security environment, diplomatic engagement within health also serves a deterrent function: it embeds national systems in multilateral arrangements that increase the political, economic, and strategic costs of interference [28–30]. For smaller health systems, such interdependence can help mitigate vulnerabilities associated with limited domestic redundancy.

In this context, deterrence operates by reducing the incentives and opportunities for adversarial or opportunistic actors—whether state or non-state—to exploit health-system vulnerabilities, such as supply-chain disruptions, medicine shortages, or capacity constraints, for strategic or destabilising purposes.

By embedding health systems within transparent, rule-based, and mutually reinforcing governance frameworks, the potential impact of such disruption is reduced, thereby lowering its strategic effectiveness and attractiveness.

Sweden’s diplomatic engagement through Nordic and EU institutions already enhances its resilience. Nordic cooperative platforms, including NORDRED, cross-border emergency agreements, and joint preparedness initiatives, create mechanisms for rapid resource sharing, mutual reinforcement, and coordinated crisis response [31, 32]. The Nordic Council’s 2025 preparedness framework explicitly positions health security as a strategic priority, calling for deeper integration of medical supply chains, emergency logistics, and crisis communication [33].

EU-level engagement provides another layer of resilience. Participation in HERA strengthens early-warning capacity, coordinated procurement, and shared manufacturing incentives [9]. Engagement with the Joint Procurement Agreement facilitates access to diversified supplies during crises and supports alignment of regulatory responses. By articulating coherent positions across these platforms, Sweden increases its influence and embeds its health system within broader structures that constrain the impact of both accidental and deliberate disruptions.

For instance, a coordinated disruption targeting pharmaceutical distribution or critical medical supplies would have significantly reduced impact within an integrated Nordic–EU framework, where shared monitoring, joint procurement, and coordinated response mechanisms can rapidly absorb shocks.

This capacity to absorb and redistribute pressure across systems reduces the potential gains from disruption, thereby contributing to deterrence.

## Hospital capacity, waiting lists, and preparedness constraints

Hospital surge capacity represents a core element of health-system resilience. Sweden’s ICU bed availability remains below many European comparators [10, 11], and elective-care waiting lists have expanded significantly since the COVID-19 pandemic, reducing operational flexibility during emergencies [12, 13]. Nordic scenario modelling indicates that simultaneous regional crises, such as pandemics, major cyberattacks, or mass-casualty events, could lead to rapid ICU saturation across multiple countries, limiting the effectiveness of bilateral patient-transfer mechanisms [22, 23].

These capacity constraints become strategically relevant when viewed through the lens of hybrid threat analysis. Adversarial actors may exploit delays, bottlenecks, or perceived weaknesses in emergency care to fuel disinformation, undermine trust, or strain public institutions [24–26]. Embedding Sweden’s hospital capacity within Nordic and EU preparedness systems helps counter these risks by expanding surge options and enabling rapid redistribution of pressures.

Regional integration thus functions as both a resilience multiplier and a deterrence mechanism: by increasing surge capacity, coordination, and system redundancy, it reduces the potential impact and visibility of disruption, thereby limiting the strategic incentives for actors seeking to exploit health-system constraints.

## Coordinated governance within Sweden

Nordic evidence consistently shows that integrated governance strengthens crisis responsiveness [33, 34]. Sweden’s ministerial structure, which distributes responsibilities for health care and social affairs, offers an opportunity, not an obstacle, for coordinated policymaking. When health policy is aligned with national security, digital infrastructure, industrial development, financial planning, and civil preparedness, the country can pursue long-term investments in surveillance systems, pharmaceutical manufacturing, AMR containment, cybersecurity safeguards, and emergency logistics [35–38].

A coherent national health-security strategy enhances the credibility and influence of Sweden’s diplomatic engagement. It ensures consistent positions across ministries and increases the likelihood that national priorities will be reflected within Nordic and EU frameworks.

Furthermore, internal coordination strengthens deterrence by reducing exploitable vulnerabilities and improving the system’s ability to anticipate, absorb, and adapt to disruptions; in doing so, it limits the potential impact and strategic value of interference, thereby reducing incentives for adversarial actors to target health-system weaknesses.

## Concerns and critiques of securitising health diplomacy

Framing health diplomacy as a component of national security and deterrence remains a contested move within global health scholarship. A substantial body of literature has cautioned against the risks associated with the securitisation of health, warning that security-oriented framing may distort public health priorities, legitimise exceptional measures, and marginalise equity-based approaches. Critics argue that when health is subsumed under national security agendas, cooperative norms risk being displaced by strategic competition, particularly in a global context characterised by widening geopolitical tensions. This concern is closely aligned with securitisation theory as developed by the Copenhagen School, which conceptualises securitisation as the elevation of issues beyond normal political processes into the realm of emergency action.

One frequently cited concern is the potential militarisation of health policy. Securitised narratives may encourage the involvement of defence actors in domains traditionally governed by civilian health authorities, thereby blurring institutional boundaries and weakening accountability. In fragile or conflict-affected settings, such dynamics have historically undermined trust in health systems and compromised humanitarian neutrality. From this perspective, extending security logics into health diplomacy risks eroding the foundational principles of global public health cooperation.

A second critique relates to global equity and power asymmetries. Health diplomacy framed through deterrence may be perceived as privileging the interests of high-income countries, particularly smaller but geopolitically embedded states, at the expense of lower-income regions. Where resilience strategies rely on regional blocs, joint procurement power, or preferential access mechanisms, there is a risk that existing inequalities in access to medicines, vaccines, and medical technologies may be exacerbated. These concerns are especially salient in light of the COVID-19 pandemic, during which global solidarity was often overshadowed by national and regional stockpiling practices.

A further objection concerns the politicisation of public health. Security framing may amplify the use of health-related disruptions as instruments of political signalling, increasing the likelihood that shortages, system stress, or epidemiological data are interpreted through adversarial lenses. Paradoxically, this may heighten rather than reduce vulnerability by rendering health systems more visible targets in disinformation campaigns or hybrid operations.

These critiques are substantial and must be taken seriously. However, they do not invalidate the strategic role of health diplomacy when carefully defined and institutionally constrained. Crucially, deterrence in this context does not imply coercion, militarisation, or zero-sum competition. Rather, it operates through institutional interdependence, transparency, and collective preparedness, increasing the political and practical costs of interference while preserving cooperative norms.

In the Swedish and Nordic context, deterrence emerges not from unilateral action but from deepened integration within civilian, rules-based frameworks. Nordic preparedness cooperation, EU joint procurement mechanisms, and shared surveillance and early-warning systems function by reducing opacity, aligning incentives, and distributing risk. These arrangements remain governed by public health authorities and regulatory agencies, rather than military command structures, and are anchored in principles of proportionality, accountability, and civilian oversight.

Moreover, embedding health systems within multilateral governance structures can reinforce, rather than undermine, equity when such frameworks are explicitly linked to transparency obligations, solidarity provisions, and shared contingency planning. At EU level, mechanisms such as joint procurement and coordinated manufacturing incentives aim to stabilise markets and prevent unilateral hoarding, thereby reducing competitive dynamics that may disadvantage smaller states.

From this perspective, health diplomacy as deterrence should be understood as a defensive and stabilising strategy, rather than a departure from global health values. It reflects the recognition that, in an era of globalised supply chains, hybrid threats, and systemic interdependence, vulnerability itself can be strategically exploited. Reducing that vulnerability through cooperation, governance alignment, and shared preparedness enhances both national resilience and collective security. By limiting the potential impact and strategic value of disruption, such approaches also reduce the incentives for adversarial actors to exploit health-system weaknesses, thereby reinforcing the deterrence function of interdependence-based governance.

Sex and gender considerations were not explicitly analysed in this debate article, as the focus is on system-level governance, pharmaceutical supply chains, and diplomatic mechanisms rather than differential clinical outcomes or access patterns. While antibiotic shortages and broader health system disruptions may have gendered implications, particularly in relation to care roles and service utilisation, these dimensions fall outside the scope of the present analysis and warrant dedicated future research.

## Conclusion

Sweden’s health system faces a complex convergence of risks, including pharmaceutical shortages, global supply-chain volatility, antimicrobial resistance, ICU capacity constraints, extended waiting lists, and hybrid threats. Addressing these challenges requires a strategy that integrates health policy with national security and positions health diplomacy at the centre of Sweden’s resilience architecture. By strengthening domestic governance coordination and embedding its health system within Nordic and European Union preparedness frameworks, Sweden can transform health diplomacy into both a cooperative instrument and a strategic asset.

Recent EU-level agreement on pharmaceutical legislation, including incentives for priority antibiotics, underscores that antibiotic security is increasingly recognised as a core component of European health resilience and strategic autonomy. This shift reflects a broader transition in which health systems are no longer viewed solely as providers of care, but as critical infrastructures essential to societal stability and security.

For Sweden, the policy implication is clear: resilience cannot be achieved through domestic capacity alone, but depends on sustained investment in multilateral coordination, shared preparedness, and governance integration. Strengthening early-warning systems, joint procurement mechanisms, and coordinated response capacities within Nordic and EU frameworks enhances not only the ability to manage disruption, but also the capacity to prevent it from becoming strategically effective.

While concerns regarding the securitisation of health diplomacy remain valid and require continuous scrutiny, neglecting the strategic dimensions of health-system vulnerability carries its own risks. A carefully bounded, governance-driven approach to health diplomacy allows Sweden to reconcile cooperation with resilience, ensuring that deterrence operates through interdependence rather than confrontation. By reducing the potential impact and visibility of disruption, such an approach limits the incentives for adversarial actors to exploit health-system weaknesses, thereby reinforcing the deterrence function of integrated health governance.

## Data Availability

No datasets were generated or analysed during the current study.
